# Assessing the scalability of a health management-strengthening intervention at the district level: a qualitative study in Ghana, Malawi and Uganda

**DOI:** 10.1186/s12961-022-00887-2

**Published:** 2022-07-30

**Authors:** Susan Bulthuis, Maryse Kok, Olivier Onvlee, Tim Martineau, Joanna Raven, Freddie Ssengooba, Justine Namakula, Hastings Banda, Patricia Akweongo, Marjolein Dieleman

**Affiliations:** 1 KIT Royal Tropical Institute, Amsterdam, The Netherlands; 2 Athena Institute, VU University, Amsterdam, The Netherlands; 3 Liverpool School of Tropical Medicine, Liverpool, UK; 4 Makerere University School of Public Health, Kampala, Uganda; 5 Research for Equity and Community Health (REACH) Trust, Lilongwe, Malawi; 6 Department of Health Policy, Planning & Management. School of Public Health, College of Health Sciences, University of Ghana, Legon, Accra, Ghana

**Keywords:** Scalability, Scale-up, CORRECT attributes, Management-strengthening intervention, Ghana, Malawi, Uganda

## Abstract

**Background:**

The scale-up of successfully tested public health interventions is critical to achieving universal health coverage. To ensure optimal use of resources, assessment of the scalability of an intervention is recognized as a crucial step in the scale-up process. This study assessed the scalability of a tested health management-strengthening intervention (MSI) at the district level in Ghana, Malawi and Uganda.

**Methods:**

Qualitative interviews were conducted with intervention users (district health management teams, DHMTs) and implementers of the scale-up of the intervention (national-level actors) in Ghana, Malawi and Uganda, before and 1 year after the scale-up had started. To assess the scalability of the intervention, the CORRECT criteria from WHO/ExpandNet were used during analysis.

**Results:**

The MSI was seen as credible, as regional- and national-level Ministry of Health officials were championing the intervention. While documented evidence on intervention effectiveness was limited, district- and national-level stakeholders seemed to be convinced of the value of the intervention. This was based on its observed positive results regarding management competencies, teamwork and specific aspects of health workforce performance and service delivery. The perceived need for strengthening of management capacity and service delivery showed the relevance of the intervention, and relative advantages of the intervention were its participatory and sustainable nature. Turnover within the DHMTs and limited (initial) management capacity were factors complicating implementation. The intervention was not contested and was seen as compatible with (policy) priorities at the national level.

**Conclusion:**

We conclude that the MSI is scalable. However, to enhance its scalability, certain aspects should be adapted to better fit the context in which the intervention is being scaled up. Greater involvement of regional and national actors alongside improved documentation of results of the intervention can facilitate scale-up. Continuous assessment of the scalability of the intervention with all stakeholders involved is necessary, as context, stakeholders and priorities may change. Therefore, adaptations of the intervention might be required. The assessment of scalability, preferably as part of the monitoring of a scale-up strategy, enables critical reflections on next steps to make the intervention more scalable and the scale-up more successful.

## Background

The scale-up of successfully tested public health interventions is critical to achieving universal health coverage in low- and middle-income countries [1, 2]. According to WHO/ExpandNet [3], scale-up can be defined as “deliberate efforts to increase the impact of successfully tested pilot, demonstration or experimental projects to benefit more people and to foster policy and programme development on a lasting basis” (p. 2). Scaling up is complex, as it “occurs across diverse systems and contexts with no one-size-fits-all approach” [4, p. 1]. Not all interventions that have been successful at a small scale are suitable for scale-up, and scaling up does not always succeed [5]. Certain intervention attributes can facilitate (or hinder) scale-up. A recent literature review identified the factors of simplicity, acceptability, relevance, effectiveness, alignment with existing systems, sustainability and adaptability of public health interventions as facilitators of scale-up [6]. To ensure optimal use of often limited resources, assessment of the scalability of an intervention is recognized as a crucial step in the scale-up process [7]. Ideally, this should take place during the pilot phase as well as during the scale-up process [8]. Milat et al. [7] define scalability as “the ability of a health intervention shown to be efficacious on a small scale and/or under controlled conditions to be expanded under real world conditions to reach a greater proportion of the eligible population while retaining effectiveness” (p. 289).

In the literature, a range of frameworks and guidance documents on scale-up highlight the importance of the scalability of an intervention and/or identifying scalable attributes of an intervention [7, 9–11]. In some frameworks, assessing scalability focuses mostly on the intervention (as a first step in the scale-up process) [9, 11], whereas in other frameworks the focus of scalability goes beyond the intervention to also focus on the scale-up process [7]. Practical tools for the assessment of the scalability of an intervention, however, are limited [12]. WHO/ExpandNet [3] developed a tool, supported by a checklist [13], to assess the scalability of an intervention as the first of nine steps necessary for the development of a scale-up strategy. The better the intervention attributes meet the CORRECT [credibility, observability, relevance, relative advantage, ease of installation and understanding, compatibility and testability] criteria, the more scalable the intervention is and the more likely it is to be successfully scaled up [3]. Reflections around scalability can assist in identifying recommendations for a scale-up strategy and how to strengthen or amplify characteristics of the intervention to increase the potential for success during scale-up. The CORRECT criteria, based on substantial literature and field research from Glaser et al., are strongly aligned with the scalability tools of Cooley et al. [9] and Cooley and Linn [14], and are further operationalized for this study in Table 2.

In the PERFORM2Scale project, a district-level health management strengthening intervention (MSI) is being scaled up in Ghana, Malawi and Uganda. The MSI aims to strengthen management competencies at the district level, a need that has been highlighted in many contexts [15–18], to ultimately improve health workforce performance and service delivery. In each country, the intervention started in three districts and is gradually scaled up to six and later nine districts over a period of 3 years. The MSI uses a participatory action research cycle [19]. Project country research teams (CRTs) facilitate district health management teams (DHMTs) in executing the plan, act, observe and reflect steps of the action research cycle. Key principles of the MSI are that DHMTs identify priority problems in their districts and that there is no financial support provided by the project to implement the activities that have been identified to address these problems throughout the cycle. More details about the MSI are provided in Box 1. To enable the scale-up of the MSI, based on guidance documents from WHO/ExpandNet [3, 13], a national scale-up steering group (NSSG) and a resource team (RT) were established. The NSSG consists of several senior staff members from the Ministry of Health (MoH), the Ministry of Local Government or faith-based organizations, depending on the country context. The RT is a team of four to eight district, regional and national management-level staff (depending on the country context) that facilitates the implementation and scale-up of the intervention, and that ultimately takes over the role of the CRT.
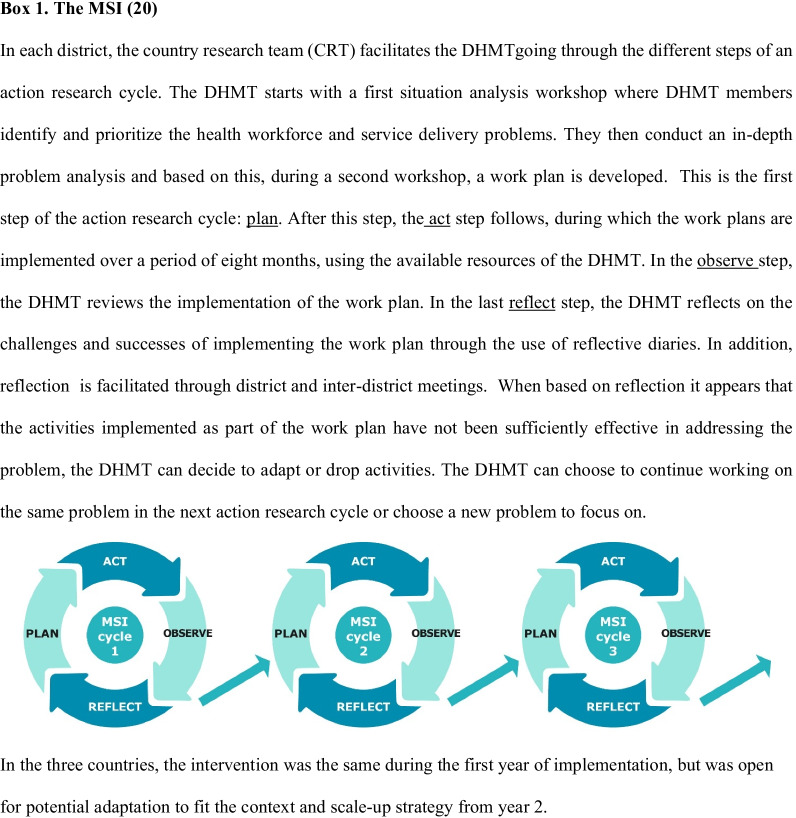


Prior to PERFORM2Scale, the MSI was piloted in Ghana, Uganda and Tanzania during the PERFORM research project (2011–2015). This study provided evidence that the MSI was effective in strengthening DHMTs’ management competencies to improve health workforce performance and service delivery [15]. Furthermore, DHMT members found that the MSI was acceptable and fit within their working commitments, in particular because they were able to identify problems and strategies in their own context [15]. Based on the PERFORM pilot, the European Union and the ministries of health in Ghana, Malawi and Uganda expressed their support for the scale-up of the MSI.

The scalability of an intervention must be assessed before and during the course of the scale-up process, because intervention attributes can change over time [8], especially those that are related to the context in which the intervention is embedded. Continuous scalability assessments can inform actions that need to be taken, for example adaptation of intervention attributes, to strengthen the scalability of the intervention and steer its scale-up. Whether an intervention is relevant, easy to install and understand, and compatible is dependent on the existing problems, priorities, norms, values and beliefs in a certain context. Assessing the scalability of an intervention is therefore challenging, and there is limited guidance on how to perform comprehensive assessments [12]. PERFORM2Scale used the CORRECT criteria because of their practicality and complementarity with the ExpandNet framework [3], which was used as guidance throughout the scale-up of the MSI. This article presents the results of a study that aimed to assess the scalability of the MSI and focused on the analysis of its (CORRECT) attributes, based on the perspectives and experiences of the users of the MSI (DHMTs) and implementers of the scale-up of the MSI (CRTs, RTs and NSSGs) in Ghana, Malawi and Uganda before and 1 year after the scale-up had started.

## Methods

We used qualitative methods to gain an in-depth understanding of the scalability of the MSI. We used data from broader studies that were conducted during two phases of the project implementation. Prior to the implementation and scale-up of the MSI (late 2017/early 2018), an initial context analysis was performed, where interviews with future users of the MSI and implementers of the scale-up of the MSI explored their understanding of the MSI and recommendations (Table 1). Participants were purposefully selected [21]. Using a topic guide, the interviews were conducted by researchers from the School of Public Health (Ghana), Reach Trust Malawi (Malawi) and Makerere University School of Public Health (Uganda), and took approximately 90 minutes.

**Table 1:** Overview of participants in Ghana, Malawi and Uganda

	Initial context analysis	Process evaluation	
	Interviews	DHMT interviews	Scale-up assessment
Ghana	MoH & GHS: 6 RHA: 2 DHMT members: 15 District assemblies: 6	9 DHMT members	3 NSSG (group discussion) 2 GHS 1 CHAG 3 RT (group discussion) 3 RHA
Malawi	MoH: 2 MoLG:3 DHMT members: 11 District council: 8	9 DHMT members	1 NSSG (individual) 1 MoH 2 RT (individual) 2 MoH
Uganda	MoH:1 MoLG: 1 Health Service Commission: 1 DHMT members: 13 Admin actors at district level: 4 Political actors at district level: 3	9 DHMT members 1 Human resource officer	3 RT members (individual) 2 MoH 1 Public Service Commission

*CHAG* Christian Health Association of Ghana, *DHMT* district health management team, *GHS* Ghana Health Service, *MoH* Ministry of Health, *MoLG* Ministry of Local Government, *NSSG* national scale-up steering group, *RHA* regional health administration

Between May and August 2019, data were collected as part of the process evaluation that aimed to obtain an in-depth understanding of how the MSI and scale-up had been implemented in each country so far, by whom and what factors were of influence. Two methods were applied (Table 1). First, to acquire insights into experiences of DHMTs, in-depth interviews were conducted with three DHMT members in each of the three districts where the MSI was implemented. Participants were purposefully selected [21]. An interview guide was used, which included reflective questions, amongst others, about the scalability of the MSI. Interviews took approximately 2 hours. Second, a scale-up assessment was performed to generate insights from national stakeholders involved in the scale-up of the MSI, on how the scale-up operated and what factors were of influence. The scale-up assessment in Ghana entailed one group discussion with NSSG members and one with RT members. Due to the busy schedules of NSSG and RT members in Malawi and Uganda, it was not possible to hold group discussions, and therefore, individual interviews were held. During the group discussions and interviews, participants individually scored statements about the scale-up process, including statements on the MSI, and subsequently discussed reasons for their scores. An interview guide was used during the group discussions and interviews. The group discussions took approximately 2 hours and the interviews 1.5 hours.

In Malawi and Uganda, the DHMT interviews and the scale-up assessments were conducted by researchers from the Royal Tropical Institute (KIT) and in Ghana by KIT and trained research assistants from the School of Public Health, University of Ghana, as those researchers had not been involved in the implementation and scale-up of the intervention.

Interviews and group discussions from the initial context analysis and process evaluation were recorded, transcribed verbatim and anonymized. In addition, detailed notes were taken during the interviews and group discussions, and the research teams held daily debriefings to discuss main findings. Thereafter, a deductive and inductive coding approach was undertaken, using QSR NVivo 11 software. While the data focused on all (potential) facilitators of and barriers to scale-up of the MSI, for the purpose of assessing the scalability of the MSI, a coding framework based on the CORRECT criteria was used, according to the operationalization provided by WHO/ExpandNet [3] (see Table 2). A matrix was developed with the key findings per CORRECT criteria and this was extensively discussed and validated by consortium partners. After this, narratives were developed according to the different CORRECT criteria, using an iterative process.

**Table 2:** CORRECT criteria

Credibility	Whether the MSI is *credible* entailed perceptions about whether or not the development of the MSI was based on sound evidence and/or whether respected persons or institutions have advocated for the MSI
Observability	The *observability* criteria focused on whether key stakeholders could see the results of the MSI in practice
Relevance	The *relevance* criteria included whether the MSI addressed a need or persistent problem as perceived by the participants
Relative advantage	The *relative advantage* criteria related to whether the MSI was perceived to have an advantage over other management-strengthening interventions
Ease of installation and understanding	Whether the MSI was *easy to install and understand* focused on whether the MSI was regarded as easy or complicated to implement in new districts and what degree of changes in current practices and human and financial resources would be necessary to enable implementation
Compatibility	The *compatibility* criteria explored whether the MSI was perceived to be compatible with the current norms, values and views of the different stakeholders
Testability	The *testability* criteria focused on whether or not respondents felt that the Ministry of Health could introduce the MSI in stages without fully adopting it

Ethical approval was provided by the Liverpool School of Tropical Medicine Research Ethics Committee, the Ghana Health Service Ethics Review Committee, the Uganda National Council for Science and Technology, the ethics committee of the School of Public Health (Makerere University) and the National Commission for Science and Technology in Malawi. Participants provided written consent before participating in the interviews and/or group discussions and they were conducted at a place where privacy could be assured. Permission for recording was requested, and full confidentiality and anonymity was ensured during the management, storage, analysis and presentation of the data.

## Results

The findings on the scalability of the MSI are presented according to the different CORRECT criteria. Where data allow, first context analysis findings (phase 1) are presented followed by findings from the process evaluation (phase 2). For the criteria “observability” and “easy to install and understand”, only process evaluation data are described, as implementation of the MSI was necessary to thoroughly reflect upon those criteria.

### Credibility

Before the start of the implementation/scale-up of the MSI, several participants at the district and national levels from each country believed that the MSI was a good intervention and acknowledged that it should be scaled up. However, participants’ notions of the credibility of the MSI were mostly based on interviewers’ explanations about what the MSI is and how it can contribute to improved district management, workforce performance and service delivery. The participants were not aware of documented evidence on the effectiveness of the MSI. One participant in Malawi mentioned that the MSI is credible because it has been successful in other countries.

*So why only choosing three districts? And if the programme has worked elsewhere (in Uganda, Ghana and Tanzania), then why not just rolling it out here, as it has already proven elsewhere that it has got advantageous on the health sector?* (District assembly participant, Malawi, phase 1)

In contrast, another Malawian participant mentioned that a pilot in Malawi needs to take place first, before something could be said about the credibility of the intervention.

One year into the implementation of the MSI, many DHMT members in Ghana and Malawi attached the intervention’s credibility to people that steered and championed the intervention, and most of those people were at a higher hierarchical level. In Ghana, several DHMT members identified the regional director, who was part of the RT as well as the NSSG, as a champion who was strongly advocating for the MSI.

*That woman is a hardworking woman and is very motivated by PERFORM2Scale than anyone I have ever seen [laughs], mainly because of the results it yielded, so she is the main champion for the PERFORM2Scale project.* (DHMT member, Ghana, phase 2)

In Malawi, several participants at the district and national levels mentioned that the Quality Management Directorate of the MoH was actively advocating for the MSI, which positively influenced its credibility. In Uganda, no specific champions emerged after 1 year of implementation.

Similar to when the intervention started, 1 year into implementation, district and national participants did not know whether the MSI was based on evidence. Only one participant, at the district level in Ghana, stated that the intervention was evidence-based, but did not provide more details.

### Observability

One year into the implementation of the MSI, the results of the MSI that were seen in practice by the different participants varied: from improved management competencies and teamwork at the level of the DHMT, to improved health worker performance and service delivery. The latter were strongly dependent upon the type of problems the DHMTs had prioritized during the action research cycle. The DHMT members discussed the changes that they had observed in greater detail compared with RT and NSSG members. The RT and NSSG members noted more general improvement and progress in the functioning of DHMTs.

#### DHMT management competencies

Many DHMT members across different districts in the three countries observed that the MSI had strengthened their management skills. In Ghana and Malawi, DHMT participants reported that they had strengthened their problem-solving capacity, resulting in better and more proactively addressing the issues that affected the implementation of DHMT activities. Furthermore, several Ghanaian DHMT members mentioned that their attitude towards work had changed and that they were now more committed to work. For example, one participant mentioned that the DHMT was no longer dependent on external resources or funding, but that they were now looking within their own means to find solutions. In Uganda and Malawi, several DHMT members explained that they had strengthened specific management skills that related to the action research cycle such as analysing problems, planning, the use of data and reflection.

*I think I can analyse my problems better because of this project; secondly, I am able to reflect on what just happened or what I just did, and because of this, initially I would not sit down and reflect, so I have seen some changes.* (DHMT member, Malawi, phase 2)

#### Teamwork and collaboration

In all three countries, several DHMT members across different districts shared their observations that teamwork within the DHMT had improved. Reference was made to better working together, more frequent communication, having a more open environment to share ideas, improved relationships among staff, improved team spirit and better interaction among units.

*Teamwork has also improved in a way, reason being that people have learnt that they cannot achieve this as individuals, and for them to perform better they have to work as a team.* (DHMT member, Uganda, phase 2)

Particular reference was made to stronger and more frequent communication between the different DHMT members in Malawi.

Some DHMT members in Ghana and Uganda mentioned that collaborations with actors outside the DHMTs, such as subdistrict staff and nongovernmental organizations (NGOs), had strengthened. For example, in Uganda, several DHMT members noted that through the MSI focus on addressing human resource management issues, such as absenteeism, the interactions and cooperation with the human resource office at the district had improved and that, as a result, the human resource office had been able to support certain activities.

*It was exciting because I think PERFORM2Scale gave us an opportunity to directly interact with the human resource office on issues of human resource for health (…) PERFORM2Scale has now taught us (…) that nothing can be done without consultation of either of the parties.* (DHMT member, Uganda, phase 2)

#### Health worker performance

In Ghana and Uganda, many DHMT participants noted that there was a reduction in absenteeism at the facility level after finalization of the action research cycle which aimed to reduce absenteeism. Several participants from two different districts in Uganda reported that absenteeism had decreased from 35 to 25%. One participant explained that unauthorized absenteeism had decreased.

*For me I would say there is change, we are progressing. You know, absenteeism is a very big thing […] we may not get the entire picture that it has reduced from this to this, but if you begin to hear from the facilities that people are now coming to the facility, that is already a sign that there is attendance to duty. So for us, even if we do not have data to track that something is taking place, we can conclude that something is taking place.* (DHMT member, Uganda, phase 2)

Furthermore, in Ghana and Malawi, several DHMT participants noted a changed attitude among health workers towards their work and/or patients after the action research cycle. This was mostly a result of increased supervision and monitoring from the DHMT members of the health facilities.

*Also [the] attitude of many workers has been improved because of rewarding or disciplinary measures. I think maybe the cleanliness as I said about the competition of infection prevention that we had whereby we were rewarding the good performers, hence the environment was clean.* (DHMT member, Malawi, phase 2)

*Yes, attitudes of staff towards clients have changed and improved as well, attitude towards work has also improved. and now they have been coming to work very early and our monitoring has confirmed that. Personally too I have seen those same changes in my attitude to work myself.* (DHMT member, Ghana, phase 2)

#### Service delivery

In Ghana, Malawi and Uganda, DHMT members also observed changes at the service delivery level. For example, in Ghana, a majority of the DHMT participants reported improvements in antenatal care coverage, as well as the yaws and buruli ulcer detection rate, as their action research cycles focused on this. In Uganda, several participants mentioned that the tuberculosis cure rate in one of the districts went up from 20 to 58% after the action research cycle. In Malawi, several participants explained that through strengthened supervision from DHMT members, the basic requirements for improved service delivery, such as water availability at the facility level and increased health workforce, had been improved.

*Firstly, it [the MSI] has helped to improve our service delivery because we have seen that in the areas where we had shortage of staff we have managed to add some more and where people were not doing things correctly they were advised. Again, we had a problem of water supply in three facilities, so this was rectified. All these problems were captured during the supervision exercise.* (DHMT member, Malawi, phase 2)

However, some other participants in Malawi stated that it was difficult to attribute any changes in service delivery to the MSI because of the presence of other projects and interventions aimed at improving the same health issues, such as maternal health.

*What we thought in our plan we could change was to reduce the maternal and neonatal deaths, of which it has been achieved, only that we are not sure on whether it’s because of PERFORM2Scale or other interventions.* (DHMT member, Malawi, phase 2)

#### How the results of the MSI were “seen” in practice (sharing of MSI effects)

Participants at the national and district levels in all three countries thought that the results of the MSI that were observed were mostly based on their own experience and observations and sometimes on “documented evidence”.

*People are convinced because, I think, basically on observation. But documentation is another area which needs to be strengthened.* (NSSG member, Malawi, phase 2)

*I really wish I could give them [other district health managers] evidence of how the project is helping us, but it is just verbal, telling them where we were and where we are now, and it is pushing us in the right direction.* (DHMT member, Malawi, phase 2)

In Malawi, the absence of documented evidence on outcomes of the action research cycles from year 1 was experienced as a problem for its scale-up by national-level stakeholders. In Ghana and Uganda, depending on the focus of the action research cycles, there was more documented evidence.

*One of the districts also identified the issue of tuberculosis treatment rates, and according to the statistics they [DHMT] showed, there is actually an improvement.* (RT member, Uganda, phase 2)

In Ghana, the NSSG and RT members explained that MSI effects were evident in the annual performance review meeting, where one of the districts presented their results on yaws case detection. The potential of using performance review meetings was also mentioned in Uganda, although it had not yet been used as an avenue to share effects of the MSI. In Ghana, NSSG members stated that meetings were held with them where the CRT presented results of actions that the DHMTs had implemented to address problems.

*Even though they were just slides and we have not read any documents from them, the slides tell the story, and the reports we get from School of Public Health complements it.* (NSSG member, Ghana, phase 2)

In all three countries, RT and NSSG members explained that results of the MSI were noted through conversations and interactions with DHMT members. An NSSG member from Ghana and an RT member from Malawi mentioned that during those conversations, DHMT members were positive about and keen to participate in the MSI.

### Relevance

During the initial context analysis interviews, several respondents in Malawi from the DHMTs and district councils made specific reference to the relevance of the MSI, as strengthening the management of the DHMTs would help DHMTs address crucial challenges at the service delivery level.

*My last words would be to acknowledge that this approach is very important because service delivery depends on the availability of a number of key factors including human and financial resources. Empowering of the human resource can assist in service delivery, because you can have the human resource and the financial resources, but if the human resource doesn’t have the capacity, then the service delivery will be poor.* (District council participant, Malawi, phase 1)

One year into the implementation of the MSI, several participants from the three countries expressed that the MSI was relevant because there was a felt need to strengthen management capacities of DHMTs. In Malawi and Uganda, some DHMT members mentioned that they were clinically trained and had only had some “on-the-job” training but were not formally trained in management.

*Because personally I am a doctor and out of medical school, all you know is medicine and patients and their treatment. So managing human resource and managing other resources is only in such [MSI] workshops that you acquire those managerial skills, so the workshops are definitely good.* (DHMT member, Uganda, phase 2)

In Ghana, a DHMT participant explained that they were taught about management cycles in school but not how to apply this to their work. Several participants at the district and national levels pointed out that DHMT members were aware of certain problems in the districts but did not have the practical skills to deal with these problems, especially not in the context of limited resources.

*PERFORM approach has really helped us, especially in the areas of problem identification process and analysis. It has equipped us with the skills and knowledge in doing this as well as looking for the strategies you can implement to solve the problem. Now that we have successfully gone through the first cycle, we can confidently say we can apply this to any problem we identify in the district. At first, we were thinking that we know that there may be yaws in the district but we do not have the funds to train our staff or do what is necessary to deal with the problem, but PERFORM has shown us the way forward with our limited resources. It is very good, and I hope other districts will get this knowledge.* (DHMT member, Ghana, phase 2)

### Relative advantage

During the initial context analysis, some participants in Ghana and Uganda mentioned that there were interventions similar to the MSI taking place in their countries, and therefore that alignment of the MSI with these interventions would be necessary. For example, in Uganda, several participants referred to the already existing Plan-Do-Study-Act (PDSA) approach or fishbone/5 Whys approaches [22] which are supposed to be used by DHMTs.

One year into implementation of the MSI, district- and national-level participants in all three countries identified three clear advantages of the MSI over similar interventions for management strengthening. First, the MSI does not come with additional resources for the DHMTs to implement activities as part of the MSI. Several DHMT members mentioned that this helps to create ownership and sustainability of the MSI. The majority of the interviewed DHMT members from the three countries mentioned the importance of the alignment of the MSI activities with the district implementation plan, as through this plan resources are made available for implementation in a sustainable way.

In Uganda, several participants were surprised that they were able to make changes using their existing resources, instead of relying on external resources.

*Because before we were used to… when a project comes we get funding from it to implement, so at first we were, “why don’t they just give us funds, why are they telling us, why can’t they just give us funds so that we are able to do more on the supervisions and all that”.* (DHMT member, Uganda, phase 2)

Second, many DHMT members from all countries identified the inter-district meetings and learning as a unique feature of the MSI. Most participants explained that the interactions with other districts enhanced exchange of ideas and learning, because the districts face similar challenges in management of district health services.

*The knowledge sharing between the three participating districts, I think that one too is the most important one. We are able to share the strategies, the knowledge, the implementation, the challenges, and so when you even listen to the other colleague from the other district then you say, “oh, maybe we also have this problem, so this is maybe some of the ways that maybe we can address them”.* (DHMT member, Ghana, phase 2)

Third, some participants from Uganda strongly appreciated the active participation and involvement of the DHMT members during the different workshops.

*I usually go for workshops, but you leave the place empty, like you participate, sometimes you just go and sit and listen to people talking, and you come back probably with your allowances and you come back to office. But the way PERFORM2Scale if it is there, their workshop is a bit different, they make sure you are part of the whole process, so... I just loved the way they do it; everyone is involved at every level from identifying the problem, the problem tree to work, they make sure everyone is involved.* (DHMT member, Uganda, phase 1)

### Ease of installation and understanding

One year into the implementation of the MSI, participants from the three countries identified (contextual) factors that influenced the ease of implementation of the MSI in new districts; these included existing planning cycles, DHMT turnover and DHMT capacity.

#### Existing planning cycles

In all three countries, several DHMT participants mentioned that integration of the MSI action research cycle into the regular health planning process is critical. This would make planning more efficient and facilitate resource allocation for MSI work plans. This did not happen in the first action research cycle, but there was better alignment in the three different countries for the second action research cycle.

*Yeah, we actually made sure we incorporate those activities into our work plan so that even when we don’t have the funding and maybe it comes on board, we can show them [NGOs/funders] that this is what we want to do this, can you help us to do this and this.* (DHMT member, Uganda, phase 2)

In Malawi, one of the DHMT members suggested that it would be better to extend the action research cycle to a 1-year cycle so that its duration is aligned with the district implementation plan cycle of the DHMTs.

#### DHMT turnover

In Ghana and Malawi, most participants at the district and national levels identified that one of the key factors complicating the implementation of the MSI was the turnover of DHMT members. The high number of staff transfers influenced the continuity of the project, as new DHMT members who replaced the previous DMHT members had not been trained on the MSI and may not have perceived it as their priority.

*One of the challenges for impact I think is that, because you get people, you train people, they start, they move out, you get new people who completely don’t know. Now at that point in time maybe people are going for mentorship, you can’t mentor someone who has no background. So that affected the programme.* (NSSG member, Malawi, phase 2)

DHMT members in Uganda did not experience transfers as a challenge in relation to the implementation of the MSI, as in Uganda the districts have more control over their staffing arrangements.

#### Capacity of DHMTs

Some participants from Malawi and Uganda mentioned that DHMT members had limited management capacity before starting with the MSI and were therefore less able to understand the MSI. For example, one DHMT member from Uganda explained that DHMTs were unfamiliar with action research projects and had difficulties in understanding the language that was used, which was referred to as “PERFORM language” but not “DHMT language”. One of the participants in Malawi identified a need for longer workshops, as management training for DHMTs was no longer taking place.

*Every manager before starting work should undergo an induction process where they could learn problem-solving, financial issues and communication. Since now they are not inducted on how to do the office work, this limited the skills which were [previously] acquired through the induction process. Therefore, I recommend a minimum of 3 weeks [for the first MSI workshop for DHMT members] will be better.* (DHMT member, Malawi, phase 2)

### Compatibility

During the initial context analysis interviews, many participants in Ghana and several participants in Malawi mentioned that the MSI aligns with the current priorities of decision-makers. Although no specific reference was made to management strengthening itself being a priority of the decision-makers, the participants perceived management strengthening as a way to address challenges in health service delivery, and therefore felt that the MSI was aligned with priorities at the national level.

*Yes, it does [fit within health priorities of decision-makers], because if you strengthen your management, it improves your efficiency, and then you can check to see if what you thought earlier can be improved to achieve another or better result.* (DHMT member, Ghana, phase 1)

A DHMT participant in Malawi explained that the MoH welcomed the MSI, as there was no person who was not interested in addressing district problems. Similar reflections were provided by a participant from Ghana: anything that was good for the health sector was a priority and would be accepted by decision-makers. However, one participant from a district council in Malawi recognized a challenge with regard to the compatibility of the MSI. There was often a tendency to prioritize those interventions that had short-term impact and not those that would take longer to have impact, such as the MSI.

*For example, if there is a management issue and cholera outbreak, the government would say, “let’s focus on cholera issues first”. This is because performance from the managerial point of view is not a short-term issue. They concentrate on short-term issues, forgetting that if you improve performance of those people on managerial skills, there will be fewer challenges. […] All this is because we are interested in issues with short-term impact, forgetting that some issues take time for the impact to be felt.* (District council participant, Malawi, phase 1)

One year into the implementation of the project, NSSG members from Ghana and Malawi explained that because the MSI is not heavily reliant on resources and/or funding, the decision-makers highly value the MSI. In Malawi, more specific reference was made to the alignment of the MSI with the existing decentralization policy. One participant from the Ministry of Local Government mentioned that because of decentralization, the functioning of DHMTs needs to be strengthened. Moreover, two RT members noted that the MSI is compatible, as it is aligned with the decentralization process, and the DHMTs can identify and work on their “own problems”. This fit of the MSI in a current policy priority was said to have resulted in key national stakeholders, such as the Quality Management Directorate of the MoH, being on board.

*I feel these stakeholders are quite convinced that this is a worthwhile programme to undertake, especially at this time [as] we are undergoing the decentralization. So they are convinced that the MSI is the way to go.* (RT member, Malawi, phase 2)

In Uganda, one RT member stated that the MSI is in line with the government’s interventions on performance management. He went on to explain that the problems and activities that were identified by the DHMTs touch the core values of the Human Resource Management Department and Public Service Commission.

*So with this MSI project, the activities which the DHMT identified, actually are in line with what the public service is all up to. […] Because in the first cycle, if I can remember, they mentioned things to do with attendance to duty, absenteeism of health workers, which do have effect on performance to districts. So that is what public service is also trying to address.* (RT member, Uganda, phase 2)

### Testability

As presented in the background section and referred to by various participants in the different countries, the MSI proved testable over the period of 2011–2015. However, for the intervention to achieve its full potential, the majority of DHMT participants in Ghana reported that it was necessary to go through the action research cycle several times (at least twice), because only then did they feel comfortable applying this approach without support.

## Discussion

This qualitative study aimed to assess the scalability of the MSI and focused on the analysis of its (CORRECT) attributes, based on the perspectives and experiences of the users and implementers in Ghana, Malawi and Uganda before and 1 year after the scale-up had started. Based on the assessment and its results, we discuss (1) reflections about the scalability of the MSI, (2) lessons learned about applying the CORRECT criteria to a complex intervention such as the MSI and (3) strengths and limitations of the study.

### The scalability of the MSI

When discussing the *credibility* of the MSI, district-level participants mostly referred to regional- or national-level actors championing the intervention. Less reference was made to the intervention being credible because its development was based on scientific evidence. It seems that the perceived credibility of the MSI in Ghana, Malawi and Uganda was linked to influential people advocating for the MSI. This might be a result of the hierarchical contexts, in particular in Ghana and Malawi [23]. This implies that the continuous involvement of regional- or national-level champions could maintain or even increase the MSI’s credibility over time, and thus enhance its scale-up.

DHMT members have been able to concretely see the results of the MSI in practice, whereas the RT members and NSSG members were able to more generally explain the results of the MSI in practice based on field visits and stories of the DHMTs. When discussing whether results of the MSI were *observable* in practice, depending on the foci of the action research cycles, DHMTs reported various outcomes, such as improved management, reduced absenteeism or increased disease case detection. While documented evidence during this implementation phase was limited, district- and national-level stakeholders seemed to be convinced of the value of the MSI based on DHMTs’ observations, which were shared during district visits and inter-district meetings.

Several scholars who focus on evidence informing health policy development have stated that there is no common definition of what evidence actually is [24, 25]. Onwujekwe et al. [25] distinguish 10 different types of evidence, which include formal and informal types of evidence, such as survey reports, systemic review reports of programmes, and proceedings from expert consultation meetings. Although formal types of evidence play a large role in policy development, expert and policy-maker opinions and experiences were identified as critical forms of evidence as well. Our study shows that this might also apply to decisions around scaling up interventions. Different stakeholders may attach more importance to different types of evidence [24], which indicates that formal documentation of the results of the MSI needs to be strengthened. In addition, reflections are necessary about who provides the evidence, as this study showed that the intervention’s credibility was often attached to who steered and/or championed the intervention, and most of those people were at a higher hierarchical level within the MoH.

In general, participants were convinced that the MSI is *relevant*, because management capacities were perceived to need strengthening and service delivery improvement was generally a goal. In particular, the direct beneficiaries of the MSI (the DHMT members) felt that the MSI is relevant, and DHMT members selecting their own problem was identified as a unique and appreciated feature of the MSI. The NSSG members (and RT members), who are supposed to adopt the scale-up of the MSI, were less outspoken with regard to the relevance. No objections were raised during the interviews about the relevance of the MSI, but because of their crucial role, it is critical that those actors also strongly feel the relevance of the MSI, which may require further action.

Several *relative advantages* of the MSI over other interventions were shared by participants, such as no provision of additional resources, the inter-district meetings and the participatory nature of the MSI. Participants made specific reference to the sustainability of the MSI, as no additional resources were provided to implement work plans. In other scalability checklists of Cooley and Linn [14] and Spicer et al. [11], sustainability of (funding for) the intervention is identified as a separate attribute of the intervention facilitating scale-up.

With regard to the MSI being *easy to install and understand*, participants identified several contextual factors that complicated the implementation and scale-up of the MSI. Some of these contextual factors are difficult to adjust to, but others can be addressed by adaptions of the MSI. For example, increasing the length of the action research cycle from 8 to 12 months could improve the embedment of the MSI in regular planning cycles, which can increase its scale-up. Kirk et al. [26] note that systematic, reactive adaptations of interventions, which are aligned with their core functions, can enhance institutionalization and thus scale-up of an intervention.

Participants in all countries said that the MSI was *compatible* in terms of priorities at the national level in relation to improving service delivery. In Malawi, specific reference was made to the MSI being compatible with the ongoing decentralization. When discussing the compatibility of the MSI, participants did not bring up issues around norms, values and interests. We could assume that the existing policy priorities at the national level that were referred to are aligned with the norms, values and interests of the relevant stakeholders. The MSI is not an intervention that is contested, as there are few or no opponents to improving district management and service delivery. Nevertheless, a more in-depth understanding of the compatibility of the MSI with the actual (institutional) norms, values, interests and arrangements could have provided relevant insight into necessary adaptations or possibilities for integration of the MSI. For example, it is important to further understand whether participatory action planning and/or reflective learning fits within the strong power dynamics based on hierarchy between the national and district levels in particular in Ghana and Malawi [23]. In the study by Svanemyr et al. [27], the compatibility of an intervention that focused on life skills-based education that included a component on sexuality education posed a challenge for scale-up, as it was not aligned with the norms, values and interests of the “conservative” society in Pakistan.

The *testability* of the MSI was hardly touched upon by the different participants, as the intervention was tested previously (2011–2015). Moreover, it is impossible to only partly implement and test a specific component of the MSI, as the intervention entails a cycle that must be passed through, and its impact is weakened when implementing only one step of the cycle.

Based on the CORRECT criteria, we conclude that the MSI is scalable; however, repeated assessment of its scalability is needed as part of the monitoring and evaluation component of the scale-up strategy in the respective countries. We also conclude that adaptations of the MSI to better fit the context can enhance its scalability, for example, by aligning the length of the MSI cycle with the district planning cycles, as well as greater involvement of regional and national champions alongside improved documentation of results of the MSI. The suggested adaptations should inform the country-specific scale-up strategies.

### Reflections on assessing interventions’ scalability

Based on our experience of applying the CORRECT criteria to the MSI, we identify several lessons learned. Before applying the CORRECT criteria, it is necessary to operationalize the CORRECT criteria in the context of the intervention that is being scaled. The criteria as presented in the WHO/ExpandNet guidance are quite broad and are related to each other or sometimes overlap, for example concerning “testability” and “easy to install and understand”. Furthermore, their operationalization depends upon the type and target population of the intervention (e.g. an MSI targeting DHMTs versus an intervention preventing gender-based violence at the household level).

The exercise of applying the CORRECT criteria to an intervention that will be scaled up should not be a “tick-box exercise”, that is, not scoring “relevant” or “not relevant”, or “scalable” or “non-scalable”. Milat et al. [12] note that limited guidance is available on how to perform a scalability assessment and that users of checklists are generally not encouraged to collect evidence to support reflections on the scalability assessment [12]. When discussing the scalability of an intervention, attributes need to be discussed and described, based on formal and informal evidence. Assessing scalability is about having a critical reflection, preferably with all stakeholders involved, on next steps to be taken to make the intervention more scalable—without the intervention “losing” its core features—and to inform the scale-up strategy to potentially make scale-up more successful. This study is therefore unique in its nature, as it has collected the perspectives of the implementers and users to assess the scalability of the MSI.

As indicated before, besides assessing the scalability of an intervention before scaling-up, it is important to assess the scalability over time, as context, stakeholders and priorities may change. Preferably, assessing the scalability of the intervention is part of the monitoring of a scale-up strategy, and based on such assessments, the attributes of an intervention can be adapted. Chambers and Norton [28] highlight the importance of continuous adaptation of interventions and note that even when evidence about its effectiveness is established for a given intervention, adaptation may still be needed. Areas for adaptation that can be considered include cultural sensitivity, mode of delivery, target audience and service setting [28]. For this particular study, the context analysis and process evaluation data have not shown a change in context or (priorities of) stakeholders, but this will be further assessed over time during a next round of the process evaluation.

We assessed the scalability of a complex intervention. Chambers and Norton state that complex interventions have “multiple interacting components, and non-linear causal pathways” [29, p. 397). Zamboni et al. [8] mention that the assessment of whether a complex intervention is scalable is challenging, because of the social processes influencing its multiple components and nonlinear pathways. When assessing the scalability of the MSI, certain attributes were indeed challenging to assess. For example, the direct results of complex health system-strengthening interventions are less easy to attribute to the intervention (observable) as compared with vertical health interventions. Interventions such as the MSI, focusing on intermediate outcomes (in this case management strengthening), may take longer to yield results on health outcomes.

While the assessment of an intervention can be seen as an important first step in the scale-up journey, scale-up requires a scale-up strategy that looks beyond the intervention and focuses on what is needed for sustained expansion and institutionalization of the intervention, in terms of dissemination, advocacy, costs and resource mobilization, and monitoring and evaluation.

### Strengths and limitations

The range of stakeholders involved during the interviews and discussions is a strength of this study and has provided different perspectives on the scalability of the MSI over time. At the time of data collection, the NSSG consisted mostly of health-related actors, such as MoH officials. As the project progressed, other stakeholders, for example from the Ministry of Local Government, became involved in the NSSG. Furthermore, group discussions were planned with the RT and NSSG members, however due to their busy schedules in Malawi and Uganda we have not been able to group the different members. Therefore, individual interviews have taken place, which has resulted in different dynamics, as less discussion between actors with different viewpoints was possible. The data for this study are derived from a broader study that focuses on factors that influence the scale-up of the MSI, one of which is the scalability of the intervention. We did not specifically focus on each CORRECT attribute in the interviews and discussions, and therefore may have missed some information on the scalability of the MSI. However, the interviews and discussions produced in-depth and rich data on participants’ views and experiences of the MSI.

## Conclusion

In this study, we have assessed the scalability of the MSI against the CORRECT attributes, and based on this assessment we can conclude that the MSI is overall a scalable intervention. Continuous assessment of the scalability over time of a complex intervention such as the MSI is necessary, as context, stakeholders and priorities may change and require adaptations of the intervention. When applying the CORRECT criteria to assess the scalability of an intervention, it is important to first operationalize the CORRECT criteria in the context of the intervention that is being scaled and to use formal and informal evidence from all stakeholders involved in the scalability assessment. Assessing scalability is not a “tick-the-box” exercise, but is about having critical reflections on next steps to be taken to make the intervention more scalable and to inform the scale-up strategy, allowing for more successful scale-up.

## Data Availability

The data that support the findings of this study are available from the corresponding author, Susan E. Bulthuis, upon reasonable request.

## Abbreviations

CHAG: Christian Health Association of Ghana
CRT: Country research team
DHMT: District health management team
GHS: Ghana Health Service
MoH: Ministry of Health
MSI: Management-strengthening intervention
NSSG: National scale-up steering group
RT: Resource team
RHA: Regional health administration
